# Determination of Phenolic Acids Using Ultra-High-Performance Liquid Chromatography Coupled with Triple Quadrupole (UHPLC-QqQ) in Fruiting Bodies of *Sanghuangporus baumii* (Pilát) L.W. Zhou and Y.C. Dai

**DOI:** 10.3390/plants12203565

**Published:** 2023-10-13

**Authors:** Zhongjing Zhou, Shuang Liang, Xiaowei Zou, Yi Teng, Weike Wang, Lizhong Fu

**Affiliations:** 1 State Key Laboratory for Managing Biotic and Chemical Threats to the Quality and Safety of Agro-Products, Zhejiang Academy of Agricultural Sciences, Hangzhou 310021, China; zhouzj@zaas.ac.cn (Z.Z.); liangs@zaas.ac.cn (S.L.); tengy@zaas.ac.cn (Y.T.); 2 College of Pharmaceutical Science, Zhejiang Chinese Medical University, Hangzhou 311402, China; zouxiaowei@zcmu.edu.cn; 3 Hangzhou Academy of Agricultural Sciences, Hangzhou 310024, China

**Keywords:** *Sanghuangporus*, *Sanghuangporus baumii*, phenolic acid, UHPLC-QqQ, quantitative

## Abstract

*Sanghuangporus*, a medicinal mushroom, has gained significant attention due to its beneficial properties. Phenolic acids are among the major bioactive compounds in *Sanghuangporus*, known for their antioxidant and anti-inflammatory activities. To precisely quantify the phenolic acid content, we developed a method utilizing ultra-high-performance liquid chromatography with triple quadrupole (UHPLC-QqQ). This study optimized the UHPLC-QqQ conditions to simultaneously separate and detect eight phenolic acids in *Sanghuangporus baumii* (Pilát) L.W. Zhou and Y.C. Dai, including chlorogenic acid, *p*-coumaric acid, caffeic acid, cryptochlorogenic acid, protocatechuic acid, ferulic acid, sinapic acid, and syringic acid. The separation process utilized a ZORBAX Eclipse Plus C18 column using 0.01% formic acid and 2 mmol/L ammonium formate in water as the aqueous phase and methanol containing 0.01% formic acid and 2 mmol/L ammonium formate as the organic phase. Calibration curves were constructed using standard solutions to quantitatively determine the phenolic acid content. The results showed significant variation in phenolic acid content among *S. baumii* fruiting bodies, with Protocatechuic acid, *p*-coumaric acid, and caffeic acid being the most abundant. This method is valuable for quantifying phenolic acid compounds under different cultivation conditions. It provides excellent sensitivity, selectivity, and reproducibility for the quantification of phenolic acids in *Sanghuangporus*, contributing to a better understanding of its chemical composition and potential health benefits. This approach represents a novel technical means for the simultaneous analysis of compound phenolic acids in *Sanghuangporus* fruiting bodies.

## 1. Introduction

Phenolic acid compounds are secondary metabolites containing phenolic hydroxyl and carboxyl groups, which are mainly produced via the phenylpropanoid pathway [1]. These compounds cannot be synthesized within the human body independently; instead, they must be obtained from external sources. With important nutritional values and biological functions, including antioxidant, anti-inflammatory, anti-microbial, cardioprotective, anticancer, and anti-diabetic properties, they have attracted much attention and are widely used in the pharmaceutical, nutraceutical, and cosmetic industries [2]. They have been employed in medicine for thousands of years due to their role in preserving the equilibrium between oxidants and antioxidants, thereby mitigating oxidative stress in the body. Recent studies have identified their beneficial skincare effects in cosmetics, such as antioxidant, anti-inflammatory, antibacterial, whitening, anti-glycation, and astringent effects [3].

While phenolic acids have been extensively studied in vegetables and fruits [2,4,5], their presence and study in mushrooms, despite their wide distribution, have received comparatively less attention [6,7,8]. Mushrooms are an excellent source of nutrients, tonics, medicines, and dietary foods that are produced and consumed all over the world. The global mushroom industry encompasses three major sectors: edible mushrooms, medicinal mushrooms, and wild mushrooms [9]. Worldwide production of mushrooms is about 40 million tonnes, with notable contributions from countries such as China, USA, Netherlands, Poland, Spain, France, Italy, Ireland, Canada, and UK [10]. The Sanghuang mushroom, known as “*Sanghuang*” in China, “*Sanghwang*” in Korea, and “*Meshimakobu*” in Japan, is one of the ten medicinal mushrooms commercially available. It belongs to the fungal phylum Basidiomycota, class Agaricomycetes, order Hymenochaetales, family Hymenochaetaceae, genus *Sanghuangporus* [11]. At present, 14 species of the genus *Sanghuangporus* have been reported worldwide [12,13], including *Sanghuangporus baumii* (Pilát), L.W. Zhou and Y.C. Dai (formerly known as *Phellinus baumii*). Modern pharmacological research has confirmed the therapeutic effects of *Sanghuangporus*, including antitumor [14], immunomodulatory [15], anti-inflammatory [16], antioxidant [11,17,18], anti-influenza [19], anti-diabetic, anti-angiogenic, radioprotective, and hypoglycemic effects [20,21].

In the past, *Sanghuangporus* was mainly obtained by people from the wild. However, due to its slow natural growth and the increasing demand for wild *Sanghuangporus*, it has been difficult for wild sources to satisfy people’s needs. Therefore, artificial cultivation of *Sanghuangporus* fruiting bodies has attracted a lot of attention since the 1990s [22]. After two to three decades of efforts, only two species, *Sanghuangporus vaninii* (Ljub.) L.W. Zhou and Y.C. Dai (formerly known as *Phellinus vaninii*) and *S. baumii*, have been successfully cultivated, and *S. baumii* is known for its rapid growth and short life cycle [17]. Both cut-log cultivation and sawdust cultivation of *Sanghuangporus* have been developed, leading to a consistent increase in fruiting body production. As production rises, the importance of quality assessment becomes evident. It is crucial to establish quality control standards for *Sanghuangporus* and to gain a more comprehensive understanding of its important bioactive components. *Sanghuangporus* is not only rich in nutrients like proteins and lipids but also contains polysaccharides, flavonoids, phenolic acids, and various other active compounds [18,23]. Among them, phenolic acid is one of the most important active components. Chien et al. reported that phenolic compounds in *Sanghuangporus* *sanghuang* have the latent ability to prevent SARS-CoV-2 infection [24].

Phenolic acids are an important category of active constituents. Their identification and quantification in *Sanghuangporus* are crucial for constructing an analysis and evaluation framework. In this study, a UHPLC-QqQ method using the MRM mode was developed and validated for the simultaneous quantification of eight phenolic acids (chlorogenic acid, *p*-coumaric acid, caffeic acid, cryptochlorogenic acid, protocatechuic acid, ferulic acid, sinapic acid, and syringic acid) in *S. baumii*, which has potential therapeutic properties. The rapidity, reliability, and sensitivity of this method were assessed, and applied to analyze eight samples of *S. baumii*. Furthermore, this method provides a strong technical support for the comprehensive quality control evaluation of *Sanghuangporus*, improves our understanding of the quantitative and qualitative analysis of various phenolic acids, and has important guiding significance for the exploration of other potential resources in *Sanghuangporus*. In addition, this method is helpful to the development and utilization of bioactive compounds in *Sanghuangporus*.

## 2. Results

### 2.1. Optimization of Parameters in the UHPLC-MS/MS Conditions

To optimize the multiple reaction monitoring (MRM) mode parameters in mass spectrometry, a syringe pump was used for sample injection at a flow rate of 7 μL/min, directly injecting a standard mixture with a concentration of 100 μg/L. The eight phenolic acids were analyzed separately in full scan in both positive [M+H]^+^ and negative [M−H]^−^ ionization modes to detect the precursor ions characteristic of each compound. First, the precursor ions were identified and then the most appropriate product ions were selected based on the fragmentation patterns of the precursor ions (Supplementary Figure S1). All analytes showed maximum sensitivity in the negative ion mode; therefore, the negative ESI-MS mode was chosen for analysis. The retention time (RT), declustering potential (DP), and collision energy (CE) values for each analyte are listed in Table 1.

In negative ion ESI-MS, all target analytes exhibited prominent [M−H]^−^ ions. Specifically, chlorogenic acid generated a precursor ion [M−H]^−^ at *m*/*z* 353.1, with fragment ions at 85.0 and 191.1 (Table 1). The product ion at *m*/*z* 191.1 was identified as quinic acid, which is formed by the cleavage of the ester bond between the quinic and caffeic acid moieties, in agreement with the literature [25]. Ferulic acid showed an [M–H]^−^ ion at *m*/*z* 193.0, a product ion at *m*/*z* 178.0 by the loss of a methyl radical, and then a product ion at *m*/*z* 134.1 through the loss of a carbon dioxide moiety (Table 1) in line with the literature. The precursor and product ions produced by other phenolic compounds were also consistent with previous studies [26,27].

**Figure 1:**
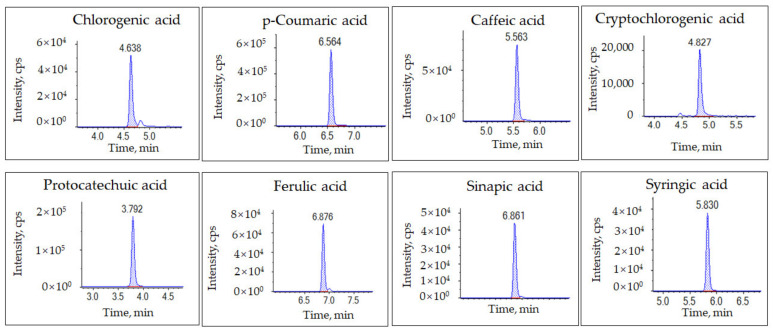
The extract ion chromatogram (XIC) of the eight phenolic acid standards (chlorogenic acid, *p*-coumaric acid, caffeic acid, cryptochlorogenic acid, protocatechuic acid, ferulic acid, sinapic acid, and syringic acid), the working concentration of each compound was 100 µg/L.

In our study, we used the extraction procedure described by Song et al. [28] and made some modifications to the phenolic acid extraction method. We used an aqueous phase containing 0.01% formic acid and 2 mmol/L ammonium formate as mobile phase A, and methanol containing 0.01% formic acid and 2 mmol/L ammonium formate as mobile phase B according to previous studies [29,30], which yield good peak shapes and low noise for the eight phenolic acids (chlorogenic acid, *p*-coumaric acid, caffeic acid, cryptochlorogenic acid, protocatechuic acid, ferulic acid, sinapic acid, and syringic acid) (Figure 1).

### 2.2. Validation of the Method

#### 2.2.1. Linearity, Limit of Detection (LOD), and Limit of Quantification (LOQ)

Mixed standard working solutions of at least five concentrations (0.05 µg/L, 0.1 µg/L, 0.5 µg/L, 1 µg/L, 5 µg/L, 10 µg/L, 50 µg/L, and 100 µg/L) were used to construct the calibration curve for each compound. The concentration was plotted on the *x*-axis while the peak area was plotted on the *y*-axis to construct the standard curve. The limit of detection (LOD) and limit of quantification (LOQ) were then calculated at signal-to-noise (S/N) ratios of 3 and 10, respectively. Detailed information on calibration curves, linear ranges, LOD, and LOQ are given in Table 2. The results indicate excellent linearity for each analyte, with coefficients of determination (R^2^) greater than 0.999. These results suggest that the current method has sufficient sensitivity for determining these compounds in the fruiting bodies of *S. baumii*.

#### 2.2.2. Precision

To assess the precision of the established method, we performed tests to evaluate intra-day and inter-day variation. For the intra-day test, samples were injected six times within a single day, while for the inter-day test, three different levels (LOQ, 2 × LOQ, and 10 × LOQ) of the mixed standard working solutions were examined over six consecutive days. The concentration of each solution was determined from a calibration curve generated on the same day. The method’s precision was evaluated by calculating the relative standard deviation (RSD) and the results are presented in Table 2. The precision values for the intra-day and inter-day tests ranged from 1.9% to 15.7% and 1.3% to 11.7%, respectively. These results indicate that our method has good repeatability and reproducibility, demonstrating its reliability for quantitative analysis.

### 2.3. Quantitative Phenolic Acids Analysis in Fruiting Bodies of S. baumii

**Figure 2:**
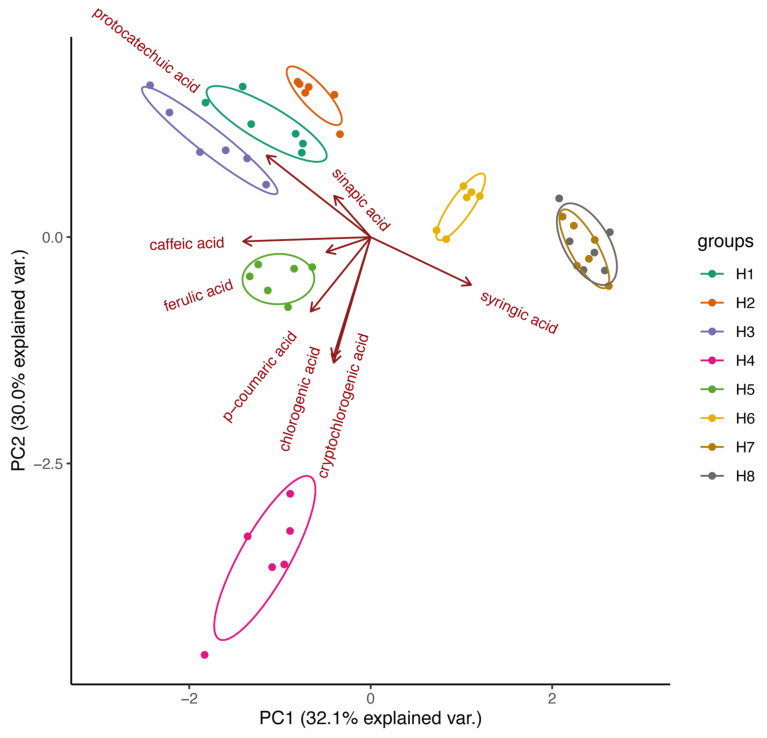
Principal Component Analysis (PCA) of the H1–H8 samples, the quantitative data of the eight phenolic acids (chlorogenic acid, *p*-coumaric acid, caffeic acid, cryptochlorogenic acid, protocatechuic acid, ferulic acid, sinapic acid, and syringic acid) were considered as variables. The arrows point to the loading plot of the variables.

The developed and validated method was applied to detect and quantify the content of eight phenolic acids in the H1–H8 samples of *S. baumii*. The Principal Component Analysis (PCA) revealed that the six replicates of the H1–H8 samples were clustered together (Figure 2). H1, H2, and H3 samples, coming from the same cut-log cultivation method, were separated from other samples on component 2. In addition, H4, H5, and H6 samples, cultivated for 30 days, 45 days, and 60 days on sawdust, respectively, are distinguishable in both components 1 and 2, indicating longer cultivation changed the phenolic acid content in fruiting bodies of *S. baumii*. However, H7 and H8 samples, cultivated for 75 days and 90 days on sawdust, respectively, are clustered together in both component 1 and component 2 (Figure 2), suggesting longer cultivation (over 75 days) did not significantly change the content of the eight phenolic compounds in the samples. And the corresponding loading plot showed the contribution of different phenolic acids to variances in component 1 and component 2. Syringic acid, caffeic acid, and protocatechuic acid were among the top three phenolic acids contributing to the variance in component 1 while cryptochlorogenic acid, chlorogenic acid, and protocatechuic acid were among the top three phenolic acids contributing to component 2. This observation indicates reproducible differences in the content of the target compounds among the samples.

**Figure 3:**
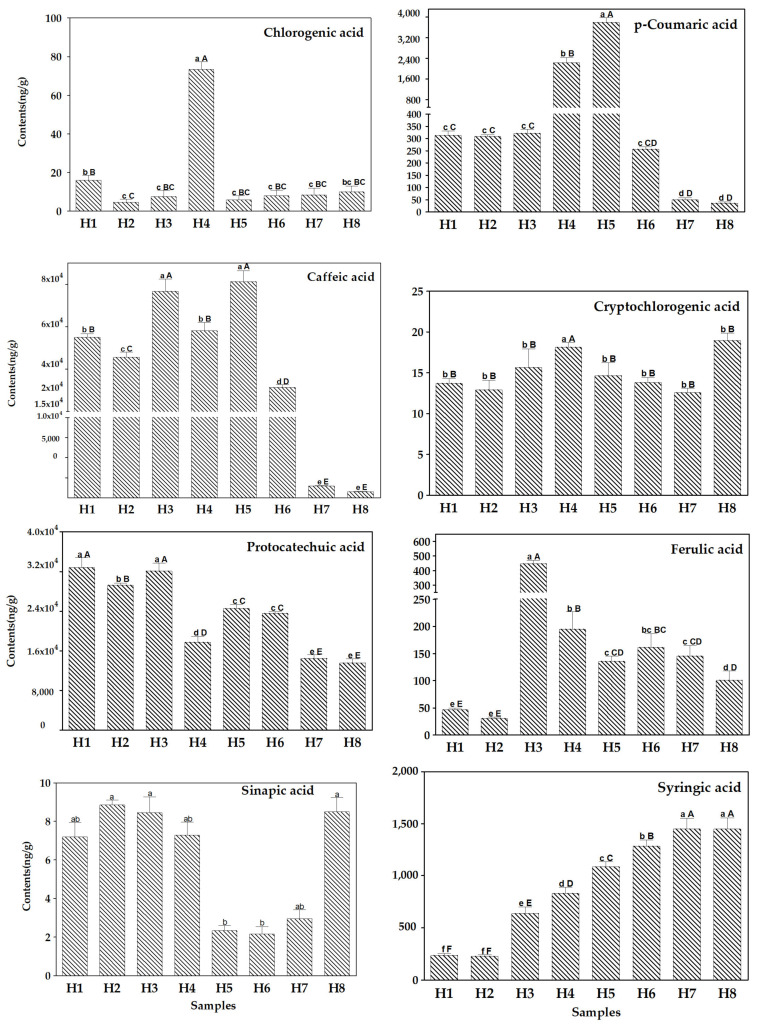
Content (ng/g Dried Weight, DW) of target analytes (chlorogenic acid, *p*-coumaric acid, caffeic acid, cryptochlorogenic acid, protocatechuic acid, ferulic acid, sinapic acid, and syringic acid) in the fruiting bodies of *S. baumii*. Values represent the samples’ mean ± SD (standard deviation, *n* = 6). Lowercase letters (a, b, c, d, e, and f) represent the significant level of 5% difference; capital letters (A, B, C, D, E, and F) indicate the very significant level of 1% difference.

Samples H1, H2, and H3, under cut-log cultivation, which are one, two, and three years old, respectively [31], did not exhibit significant differences in *p*-coumaric acid, cryptochlorogenic acid, and sinapic acid content, but showed significant differences in caffeic acid, ferulic acid, and syringic acid content. Sample H4 contained the highest levels of chlorogenic acid and cryptochlorogenic acid, and also its *p*-coumaric acid content differed from the other samples. Meanwhile, sample H5 contained the highest levels of *p*-coumaric and caffeic acids, with highly significant differences from the other samples under the same cultivation conditions (H4, H6–H8). In the H6 sample, the content of caffeic acid and syringic acid differed from that of the other samples at highly significant levels. And in H7 and H8 samples, the content of caffeic acid, protocatechuic acid, and syringic acid differed from that of other samples by highly significant levels, with the highest content of syringic acid (Figure 3). The results showed that *S. baumii* contains different amounts of phenolic acids and the content of chlorogenic acid, cryptochlorogenic acid, ferulic acid, and sinapic acid were low, but the detection and quantification by this method were reliable with good chromatographic separations, which highlights the high sensitivity of the developed method. The method provides a strong technical support for the further study of active phenolic acid markers in *S. baumii*.

## 3. Discussion

Phenolic acids are aromatic acid compounds consisting of a phenolic ring on a C6-C1 backbone, and an organic carboxylic acid functional group, which are classified into three groups according to the number of carbon chains: hydroxybenzoic acids, acetophenones/phenylacetic acids, and hydroxycinnamic acids. Hydroxybenzoic acids have a carbon chain attached to the phenolic ring, C6-C1 type, and include chlorogenic acid, cryptochlorogenic acid, protocatechuic acid, and syringic acid. Hydroxycinnamic acids have three carbon chains attached to the phenolic ring, C6-C3 type, including *p*-coumaric acid, caffeic acid, ferulic acid, and sinapic acid [32,33]. The results of the present study showed that eight phenolic acids including chlorogenic acid, *p*-coumaric acid, caffeic acid, cryptochlorogenic acid, protocatechuic acid, ferulic acid, sinapic acid, and syringic acid, were present in the fruiting bodies of *S. baumii*. Chlorogenic acid, caffeic acid, and ferulic acid show biological activities such as antioxidant, antibacterial, anti-inflammatory, antiviral, anticancer, antiaging, antidiabetic, antimicrobial, neuroprotective, cardioprotective, and antihypertensive [34,35] activities. Protocatechuic acid may protect cardiomyocytes and modulate their mechanism of action [36]. *p*-coumaric acid may control a variety of diseases, such as renal disease, cardiovascular disease, neuroinflammatory disease, liver disease, cancer, and some metabolic disorders [37]. Syringic acid has a wide range of therapeutic applications in the prevention of diabetes, cardiovascular disease, cancer, and cerebral ischemia [38]. Sinapic acid has biological activities such as antioxidant, antibacterial, anticancer, and UV-filtering activities [39]. The presence of chlorogenic acid, *p*-coumaric acid, caffeic acid, cryptochlorogenic acid, protocatechuic acid, ferulic acid, sinapic acid, and syringic acid in the fruiting bodies of *S. baumii* suggests a promising future for *Sanghuangporus* in the research and development of pharmaceuticals, nutraceuticals, and cosmetics.

The most commonly used tools for metabolite analysis are nuclear magnetic resonance spectroscopy (NMR) [40], liquid chromatography–mass spectrometry (LC/MS) [41], and gas chromatography–mass spectrometry (GC/MS) [42]. Analysis using metabolomics tools is complex in terms of separation and detection due to the intricate nature of primary and secondary metabolites found in biological samples. Metabolite analysis of biological samples can generate a large amount of data, but processing and analyzing the data is both time-consuming and challenging. Ultra-high-performance liquid chromatography (UHPLC) coupled with triple quadrupole mass spectrometry (QqQ-MS) in the multiple reaction monitoring (MRM) mode provides accurate and sensitive results suitable for quantitative analysis [30,43]. The UHPLC-MS/MS method has been applied to the determination of biologically active constituents in traditional Chinese medicines [44]. Song et al. applied a UPLC-MS/MS-based metabolomics analysis to determine the metabolic profiles of *Sanghuangporus* basidiocarps, which provided a valuable reference for the comprehensive evaluation and utilization of *Sanghuangporus* [28]. However, there is still a great need for the quantitative study of phenolic acid monomers in *Sanghuangporus* using UHPLC-QqQ.

In this study, the content of phenolic acid monomers varied significantly among samples (Figure 2 and Figure 3). The PCA results revealed a distinct separation between samples cultivated on cut-log (H1 to H3) and those on sawdust (H4 to H8), and showed the impact of varying cultivation durations on the content of phenolic acids in *S. baumii* fruiting bodies (see Figure 2). Examination of the loading data from the PCA analysis revealed that the top three variables contributing to the variance in component 1 were syringic acid, caffeic acid, and protocatechuic acid. This observation aligns with the results of the ANOVA analysis, which identified six, five, and five distinguishable levels for the content of syringic acid, caffeic acid, and protocatechuic acid in the samples, respectively (see Figure 3). For example, the content of syringic acid in samples H1, H2, and H3 was 236.8 ng/g, 226.7 ng/g, and 637.6 ng/g, respectively, suggesting that longer cultivation times could increase the syringic acid content in cut-log cultivated *S. baumii* fruiting bodies. The syringic acid contents of samples H4, H5, H6, H7, and H8 were 829.2 ng/g, 1.085 × 10^3^ ng/g, 1.283 × 10^3^ ng/g, 1.447 × 10^3^ ng/g, and 1.448 × 10^3^ ng/g, respectively, which showed that the syringic acid content increased with the increase in the cultivation period up to 75 days. In addition, the syringic acid content of these five samples was significantly higher than that of H1, H2, and H3, indicating that the cultivation method had a strong influence on the syringic acid content. Protocatechuic acid was significantly higher in the cut-log cultivated samples than in the sawdust cultivated samples, suggesting that cut-log cultivation is a better method when protocatechuic acid is desired for production. Chlorogenic acid and cryptochlorogenic acid were highest in sample H4 and differed from the other samples at highly significant levels, suggesting that early harvesting is suitable when both phenolic acids are desired during sawdust cultivation. In summary, phenolic acid monomer content can help us breed *Sanghuangporus* varieties with a high content of certain phenolic acid monomers, or determine the optimal time for harvesting certain monomers.

In this study, a rapid and sensitive UHPLC-QqQ method was developed for the simultaneous analysis of eight phenolic acid monomers in *S. baumii* fruiting bodies. The analytical method was able to rapidly detect and quantify the phenolic acids in *S. baumii* fruiting bodies with high accuracy, precision, and selectivity. Our advanced UHPLC-QqQ analytical method has several advantages over previously reported methods for the quantification of phenolic acid monomers in *Sanghuangporus*. Firstly, it can detect eight phenolic acid monomers simultaneously, making it more sensitive and selective. Secondly, by using a C18 column and optimizing the mobile phase, it has excellent separation efficiency, which is essential for the accurate quantification of eight phenolic acid monomers. Finally, our method is fast, simple, and easy to use, making it suitable for routine analysis of large numbers of samples. Our method can be used to evaluate the quality of *Sanghuangporus* fruiting bodies and be used to screen for *Sanghuangporus* varieties with higher levels of specific phenolic acid monomers. The evaluation system can also be used to guide the breeding work of *Sanghuangporus*, the improvement of cultivation technology, and the pharmacological study of *Sanghuangporus*. The results of the study showed that *Sanghuangporus* has great potential in pharmaceuticals, health products, and cosmetics.

## 4. Materials and Methods

### 4.1. Materials

The samples used in this research were fruiting bodies of *S. baumii.* H1 to H3 samples, were under cut-log cultivation, which were one, two, and three years old, respectively. For cut-log cultivation, the procedure involved soaking the oak trunk in clean water, draining surface water, placing it into a polypropylene bag, and sterilizing it in an autoclave. Following inoculation, the trunk was incubated at 25 °C in darkness for 270 days. Thirty bags were collected for each cultivation condition. After one to three years of standardized management, the fruiting bodies were harvested. H4–H8 samples were under sawdust cultivation, for 30 days, 45 days, 60 days, 75 days, and 90 days respectively, from the formation of primordia to the harvest of fruiting bodies. Sawdust cultivation followed a standard protocol: a mixture composed of 1.5 kg of mulberry branch (83%), wheat bran (15%), sucrose (1%), gypsum (1%), and water (62%) was placed into polypropylene bags and autoclaved. After the temperature dropped below 25 °C, 15 g of *S. baumii* strains were introduced, and the bags were kept at 25 °C in a dark environment. Once mycelium colonization was achieved within the substrate, the bags were transferred to an environment maintained at 25 °C with a light intensity of approximately 300 Lux for a duration of 30 days. Throughout the cultivation process, relative humidity levels were managed at 60–70% during the mycelium growth period, 90–95% during the initial primordium period, and 80–90% during the fruiting body growth period. One hundred bags were collected for each condition, and the fruiting bodies were harvested at different time points, specifically after 30, 45, 60, 75, and 90 days of growth.

### 4.2. Chemicals and Reagents

LC-MS grade methanol was purchased from Merck (Darmstadt, Germany). Formic acid was purchased from Fisher Scientific (LC-MS grade, Thermo Scientific, Carlsbad, CA, USA), and ammonium formate was purchased from Macklin (HPLC grade, purity: ≥99%). Chlorogenic acid (CAS No. 327-97-9), *p*-coumaric acid (CAS No. 501-98-4), caffeic acid (CAS No. 331-39-5), ferulic acid (CAS No. 537-98-4), sinapic acid (CAS No. 530-59-6), cryptochlorogenic acid (CAS No. 905-99-7), syringic acid (CAS No. 530-57-4), and protocatechuic acid (CAS No. 99-50-3) were purchased from Shanghai Yuanye Bio-Technology Co., Ltd. (Shanghai, China). Ultrapure water was purified using a Milli-Q system (18 MΩ × cm, Millipore, Burlington, MA, USA).

### 4.3. Instruments

The UHPLC-MS/MS system consists of a Triple Quad™ LC-MS/MS 5500+ system (SCIEX, Framingham, MA, USA) and an Ultra Performance Liquid Chromatography UHPLC, ExionLC™ AD (Shimadzu, Kyoto, Japan). Xinzhi SB25-12DTD ultrasonic bath (Ningbo, China) was used for ultrasonic-assisted extraction. Vortex-Genie 2 (Scientific Industries, Bohemia, NY, USA) was used to mix the sample solution. A heating and drying oven (Jinghong DHG-9240A, Shanghai, China) was used for drying and heat treatment of samples. An electronic analytical balance (Mettler-Toledo ME204, Zurich, Switzerland) was used to weigh the samples accurately. A medical refrigerator (Aucma YCD-265, Qingdao, China) was used to store experimental samples. A 0.22 µm syringe filter (Biosharp life sciences, Hefei, China) was used for sample filtration. Centrifugation of the sample solution was performed using a 5418 high-speed centrifuge (Eppendorf Corp., Hamburg, Germany).

### 4.4. Preparation of Standard Solutions

Each compound was accurately weighed and dissolved in methanol to prepare a standard stock solution at a concentration of 1 mg/mL. The standard working solutions of the analytes were then prepared by diluting the mixed stock solution with 70% methanol-water (*v*/*v*) to the required concentration. Standard compound solutions with different concentrations were prepared and stored in the dark in a −20 °C refrigerator for future use.

### 4.5. Sample Preparation

After harvesting, the fruit body was cut into 2–4 mm thick slices, dried in an oven at temperatures below 60 °C until a constant weight was achieved, crushed, passed through an 80-mesh sieve, and placed in an airtight polypropylene self-sealing bag. Prior to the determination of the phenolic acid content, the sample moisture content was determined by the drying method of the Pharmacopoeia of the People’s Republic of China (2020 edition). The sample pretreatment for phenolic acid determination is as follows: Six aliquots of 0.1 g *S. baumii* powder were accurately weighed and placed into 2 mL centrifuge tubes. Then 1.5 mL of 70% methanol-water (*v*/*v*) was added to each tube and vortexed for 5 min, followed by extraction in an ultrasonic bath for 30 min. The mixture was centrifuged at 12,000 rpm for 20 min at 4 °C and the supernatant was filtered through a 0.22 µm syringe filter before injection into the UHPLC-QqQ system for analysis. It should be noticed that the extraction of phenolic acid may not have been exhaustive due to the limited duration of the extraction process.

### 4.6. UHPLC Conditions

Liquid phase separation was performed on a ZORBAX Eclipse Plus C18 column (1.8 µm, 3.0 mm × 100 mm, Agilent Technologies Inc., Santa Clara, CA, USA) at a column temperature of 40 °C. The mobile phase consisted of 0.01% formic acid and 2 mmol/L ammonium formate in water (mobile phase A), and 0.01% formic acid and 2 mmol/L ammonium formate in methanol (mobile phase B). The gradient elution program was optimized as follows: 0~1.0 min, 90% A, 10% B; 1.0~11.0 min, 90% A~5% A, 10% B~95% B; 11.0~13.0 min, 5% A, 95% B; 13.0~13.1 min, 5% A~90% A, 95% B~10% B; and 13.1~15 min, 90% A, 10% B. The flow rate was 0.4 mL/min and the injection volume was 1 μL.

### 4.7. MS/MS Conditions

Mass spectrometry data were acquired using the Analyst 1.7.1 dynamic MS/MS acquisition software, and data analysis was performed using SCIEX OS software 1.7.0.36606. The ion source was operated in negative electrospray ionization (ESI^-^) mode with multiple reaction monitoring (MRM) scans. The spray voltage was −4500 V in negative ion mode and the ion source temperature was 500 °C. The pressure of ion source Gas1 (Gas1) was 50 psi, and the auxiliary gas (Ion source Gas2, Gas2) was 55 psi. The curtain gas (CUR) pressure was 35 psi and the collision gas (CAD) pressure was 8 psi. The scanning and detection parameters for each ion pair based on the optimized declustering potential (DP) and collision energy (CE) are detailed in Table 1.

### 4.8. Statistical Analysis

Quantitative data were presented as means with a standard deviation of the means from six independent experiments. Data were analyzed using Microsoft Excel. GraphPad Prism 5.0 software (GraphPad Software, San Diego, CA, USA) was used to analyze the data and construct the graphs. Principal Component Analysis (PCA) was performed using the R package ggbiplot, and analyses of variance (ANOVA) were performed through a DPS data processing system [45].

## 5. Conclusions

This study aims to achieve several specific objectives. Firstly, we seek to establish a robust ultra-high-performance liquid chromatography coupled with triple quadrupole (UHPLC-QqQ) method capable of simultaneously detecting eight different phenolic acids present in *S. baumii* samples. Secondly, our focus extends to the careful validation of this method. This involves a comprehensive assessment of key parameters including linearity, limits of detection (LOD), limits of quantification (LOQ), accuracy, and precision. By subjecting our method to rigorous validation, we are establishing its reliability and suitability for the intended analysis. Thirdly, we aim to put our method into practice. Specifically, we will use the developed UHPLC-QqQ approach to quantify the content of phenolic acids in a set of eight different *S. baumii* samples. This real-world application of our method allows us to gain valuable insights into the actual phenolic acid composition present in these samples.

In summary, the developed and validated method proved to be highly effective in detecting and quantifying phenolic acids present in *S. baumii* samples. The results provided significant insight into the relative concentrations of these compounds, highlighting that caffeic acid and protocatechuic acid have higher levels compared to other phenolic acids. The method’s exceptional sensitivity and reliable chromatographic separation make it immensely valuable in advancing research on active phenolic acid markers in *S. baumii*. This study not only improves our understanding of the chemical composition of *S. baumii*, but also provides a solid foundation for future investigations into its potential therapeutic applications.

## Figures and Tables

**Table 1:** Retention time (RT) and MRM conditions of eight phenolic acid compounds for UHPLC-ESI-MS/MS analysis.

Analyte	Q1	Q3	RT (min)	DP (V)	CE (V)
Chlorogenic acid ^1^	353.1	85.0	4.64	−74	−59
Chlorogenic acid ^2^	353.1	191.1	4.64	−56	−19
*p*-Coumaric acid ^1^	163.0	119.0	6.56	−60	−19
*p*-Coumaric acid ^2^	163.0	117.1	6.56	−71	−43
Caffeic acid ^1^	179.1	134.0	5.56	−74	−31
Caffeic acid ^2^	179.1	89.1	5.56	−60	−42
Cryptochlorogenic acid ^1^	353.1	173.0	4.83	−50	−23
Protocatechuic acid ^1^	153.1	109.0	3.79	−50	−19
Protocatechuic acid ^2^	153.1	91.0	3.79	−57	−34
Ferulic Acid ^1^	193.0	134.1	6.88	−50	−20
Ferulic Acid ^2^	193.0	178.0	6.88	−50	−16
Sinapic acid ^1^	223.0	193.0	6.86	−51	−29
Syringic acid ^1^	197.1	123.0	5.83	−41	−30
Syringic acid ^2^	197.1	181.8	5.83	−41	−17

^1,2^ refer to the first and second pairs of precursor and product ions for each compound, respectively. The first pair is for quantitative analysis, the second one for qualitative analysis.

**Table 2:** Linearity regression equation, LOD, LOQ, Intra-day, and Inter-day for the eight phenolic acids (LOD, Limit of detection; LOQ, Limit of quantification).

Analyte	Regression Equation (Weighting: 1/x)	R^2^	Linear Range (µg/L)	LOD (µg/L)	LOQ (µg/L)	Intra-Day (RSD, %) (*n* = 6)	Inter-Day (RSD, %) (*n* = 6)
Chlorogenic acid	y = 2453.62 x − 106.25	0.99957	0.10–100	0.429	1.429	5.2–9.8	1.4–6.5
*p*-Coumaric acid	y = 20240.55 x + 1604.53	0.99932	0.10–100	0.566	1.887	3.9–9.9	1.6–2.4
Caffeic acid	y = 2804.30 x + 769.49	0.99905	0.05–100	0.273	0.909	5.1–11.3	2.0–9.7
Cryptochlorogenic acid	y = 879.37 x − 388.26	0.99910	1.00–100	0.333	1.111	3.4–5.9	1.9–6.9
Protocatechuic acid	y = 5268.99 x + 3490.54	0.99929	1.00–100	0.857	2.857	4.5–10.9	2.1–5.0
Ferulic Acid	y = 2811.47 x + 1720.03	0.99944	0.50–100	0.143	0.476	2.8–13.7	1.3–7.5
Sinapic acid	y = 1697.38 x + 514.08	0.99955	0.05–100	0.333	1.111	1.9–15.7	1.7–11.7
Syringic acid	y = 1427.33 x + 150.21	0.99955	0.50–100	0.353	1.176	4.8–11.8	4.4–10.1

## Data Availability

Data is contained within the article or Supplementary Material.

## Supplementary Materials

The following supporting information can be downloaded at: https://www.mdpi.com/article/10.3390/plants12203565/s1. Figure S1: The precursor ions and selected product ions for each standard compound. The monitored precursor ion and product ion for the analytes were (A) chlorogenic acid, *m*/*z*, 353.1 > 85.0, 191.1; (B) *p*-coumaric acid, *m*/*z*, 163.0 > 117.1, 119.0; (C) caffeic acid, *m*/*z*, 179.1 > 134.0, 89.1; (D) cryptochlorogenic acid, *m*/*z*, 353.1 > 173.0; (E) protocatechuic acid, *m*/*z*, 153.1 > 109.0, 91.0; (F) ferulic acid, *m*/*z*,193.0 > 134.1, 178.0; (G) sinapic acid, *m*/*z*, 223.0 > 193.0; and (H) syringic acid, *m*/*z*, 197.1 > 123.0, 181.8. Figure S2: The total ion chromatogram (TIC) of the eight phenolic acids in the mixed standard solutions (A) and in the H4 samples (B). 1: protocatechuic acid, 2: chlorogenic acid, 3: cryptochlorogenic acid, 4: caffeic acid, 5: syringic acid, 6: *p*-coumaric acid, 7: sinapic acid, and 8: ferulic acid. The concentration of each compound in the standard solution was 100 µg/L (A). Some compounds were not visible in (B) because of their low peak intensity.
