# Supplementary material for: Determination of Phenolic Acids Using Ultra-High-Performance Liquid Chromatography Coupled with Triple Quadrupole (UHPLC-QqQ) in Fruiting Bodies of Sanghuangporus baumii (Pilát) L.W. Zhou and Y.C. Dai
Source: Plants (Basel). 2023 Oct 13;12(20):3565. doi: 10.3390/plants12203565 (PMC10609702; doi:10.3390/plants12203565)
Supplement: Supplementary file 1 [file plants-12-03565-s001.zip › Supplementary materials.pdf]

Supporting information for

Determination of Phenolic Acids Using Ultra-High-Performance Liquid Chromatography Coupled with Triple Quadrupole (UHPLC-QqQ) in Fruiting Bodies of *Sanghuangporus baumii* (Pilát) L.W. Zhou and Y.C. Dai

Zhongjing Zhou [1], Shuang Liang [1], Xiaowei Zou [2], Yi Teng [1], Weike Wang [3,*] and Lizhong Fu [2,*]

[1] State Key Laboratory for Managing Biotic and Chemical Threats to the Quality and Safety of Agro-Products, Zhejiang Academy of Agricultural Sciences, Hangzhou 310021, China; zhouzj@zaas.ac.cn (Z.Z.); liangs@zaas.ac.cn (S.L.); tengy@zaas.ac.cn (Y.T.)

[2] College of Pharmaceutical Science, Zhejiang Chinese Medical University, Hangzhou 311402, China; zouxiaowei@zcmu.edu.cn

[3] Hangzhou Academy of Agricultural Sciences, Hangzhou 310024, China

* Correspondence: akeok@126.com (W.W.); fulizhong@zcmu.edu.cn (L.F.)

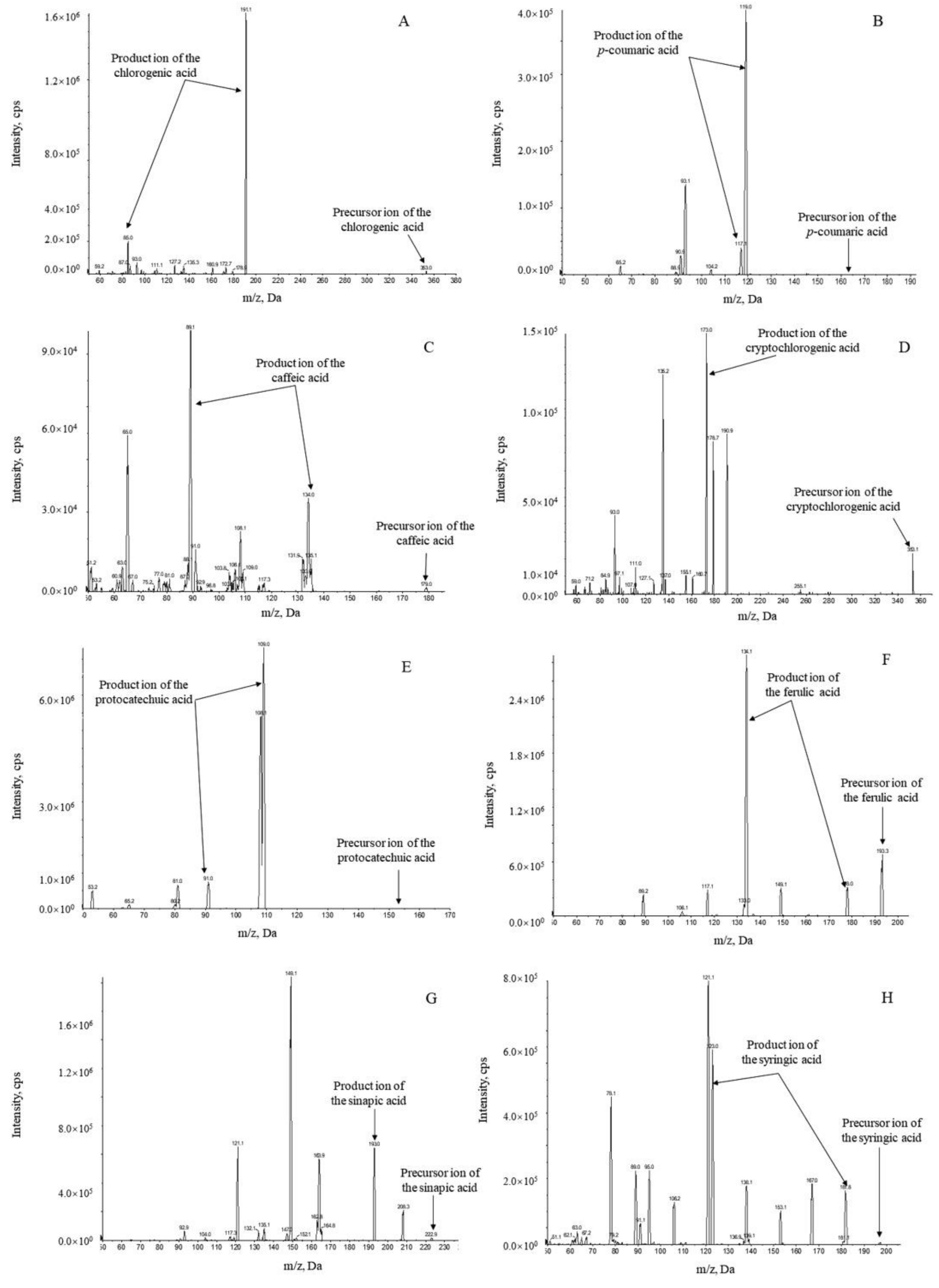

Figure Supplementary S1. The precursor ions and selected product ions for each standard compound. The monitored precursor ion and product ion for the analytes were (A) chlorogenic acid, m/z, 353.1 > 85.0, 191.1; (B) p-coumaric acid, m/z, 163.0 > 117.1, 119.0; (C) caffeic acid, m/z, 179.1 > 134.0, 89.1; (D) cryptochlorogenic acid, m/z, 353.1 > 173.0; (E) protocatechuic acid, m/z, 153.1 > 109.0, 91.0; (F) ferulic acid, m/z,193.0 > 134.1, 178.0; (G)sinapic acid, m/z, 223.0 > 193.0; and (H) syringic acid, m/z, 197.1 > 123.0, 181.8.

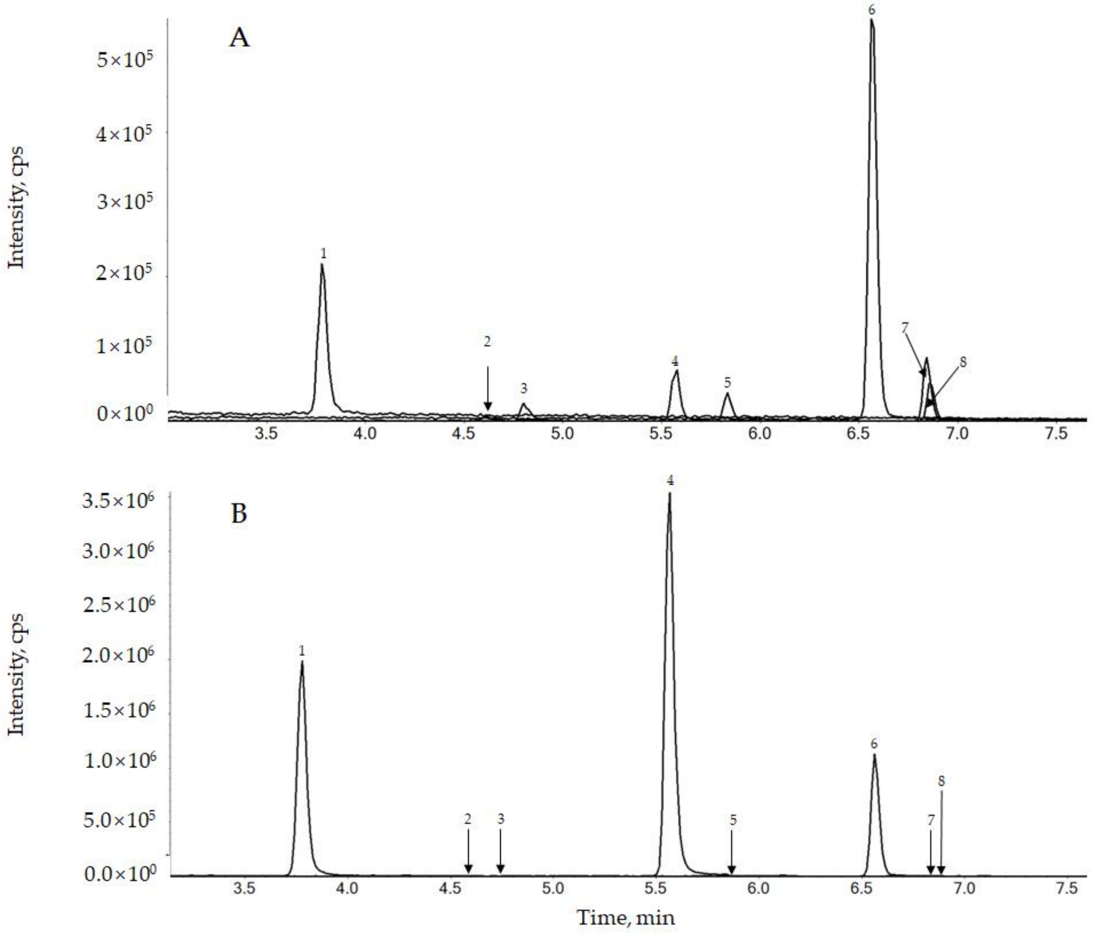

Figure Supplementary S2. The total ion chromatogram (TIC) of the eight phenolic acids in the mixed standard solutions (A) and in the H4 samples (B). 1: protocatechuic acid, 2: chlorogenic acid, 3: cryptochlorogenic acid, 4: caffeic acid, 5: syringic acid, 6: p-coumaric acid, 7: sinapic acid, and 8: ferulic acid. The concentration of each compound in the standard solution was 100 μg/L(A). Some compounds were not visible in (B) because of their low peak intensity.
