# Supplementary figures and images for: Determination of Phenolic Acids Using Ultra-High-Performance Liquid Chromatography Coupled with Triple Quadrupole (UHPLC-QqQ) in Fruiting Bodies of Sanghuangporus baumii (Pilát) L.W. Zhou and Y.C. Dai
Source: Plants (Basel). 2023 Oct 13;12(20):3565. doi: 10.3390/plants12203565 (PMC10609702; doi:10.3390/plants12203565)

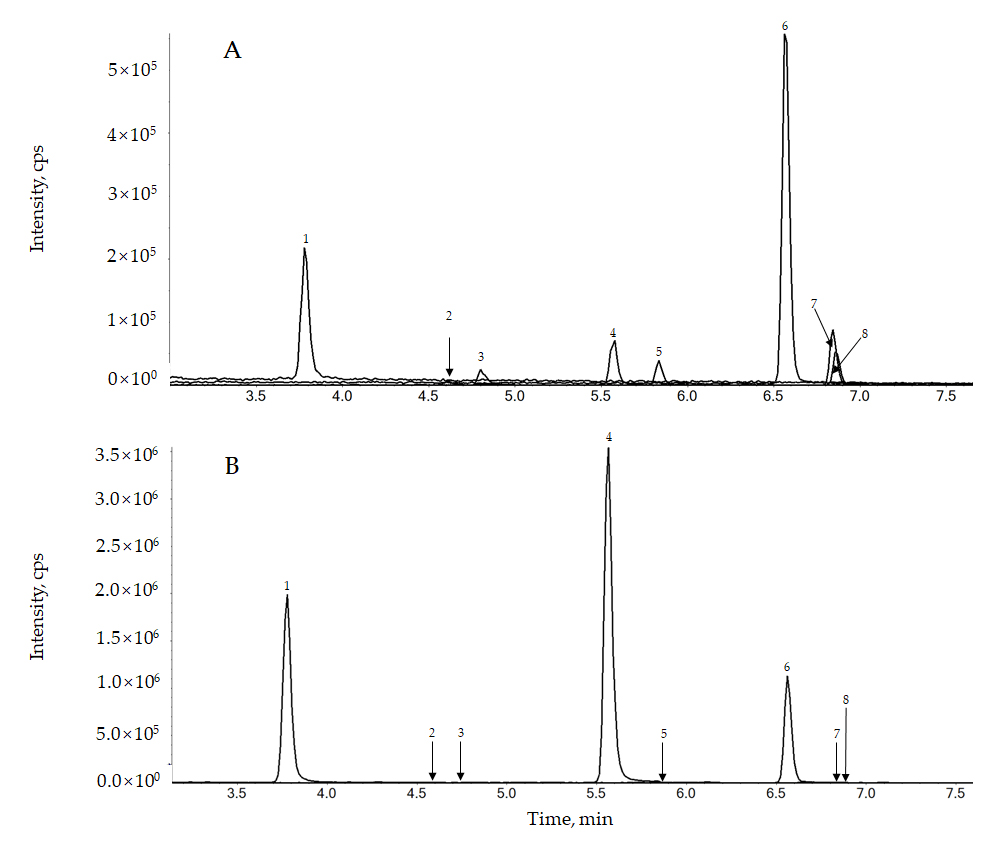

Supplement: Supplementary file 1 [file plants-12-03565-s001.zip › Figure S2.jpg]
